# Quality Attributes and Fatty Acid, Volatile and Sensory Profiles of “Arbequina” *hydroSOStainable* Olive Oil

**DOI:** 10.3390/molecules24112148

**Published:** 2019-06-06

**Authors:** Lucía Sánchez-Rodríguez, Marina Kranjac, Zvonimir Marijanović, Igor Jerković, Mireia Corell, Alfonso Moriana, Ángel A. Carbonell-Barrachina, Esther Sendra, Francisca Hernández

**Affiliations:** 1 Department of Agro-Food Technology, Research Group “Food Quality and Safety”, Universidad Miguel Hernández de Elche, Escuela Politécnica Superior de Orihuela, 03312 Orihuela, Alicante, Spain; lucia.sanchez@goumh.umh.es; 2 Department of Organic Chemistry, Faculty of Chemistry and Technology, University of Split, 21000 Split, Croatia; mkranjac@ktf-split.hr (M.K.); igor@ktf-split.hr (I.J.); 3 Department of Food Technology and Biotechnology, Faculty of Chemistry and Technology, University of Split, 21000 Split, Croatia; zmarijanovic@ktf-split.hr; 4 Departamento de Ciencias Agroforestales, ETSIA, Universidad de Sevilla, 41013 Sevilla, Spain; mcorell@us.es (M.C.); amoriana@us.es (A.M.); 5 Unidad Asociada al CSIC de Uso Sostenible del Suelo y el Agua en la Agricultura (US-IRNAS), 41013 Sevilla, Spain; 6 Department of Agro-Food Technology, Research Group “IPOA”, Universidad Miguel Hernández de Elche, Escuela Politécnica Superior de Orihuela, 03312 Alicante, Spain; esther.sendra@umh.es; 7 Department of Plant Science and Microbiology, Research Group “Plant Production and Technology”, Universidad Miguel Hernández de Elche, Escuela Politécnica Superior de Orihuela, 03312 Orihuela, Alicante, Spain; francisca.hernandez@umh.es

**Keywords:** total phenol content, oleic acid, regulated deficit irrigation, sustained deficit irrigation, antioxidants, fatty acids

## Abstract

The use of deficit irrigation techniques on olive orchards is the main trend aiming to optimize water savings while improving functional and sensory characteristics of oils from trees under deficit irrigation techniques. The brand *hydroSOStainable* has been defined for crops produced under water restriction conditions. *HydroSOStainable* olive oils obtained under two new regulated deficit irrigation and one sustained deficit irrigation treatments in “Arbequina” olive trees were evaluated by analyzing quality parameters, antioxidant activity, total phenol content, fatty acid profile, volatile compounds, and sensory descriptors. Results showed that some of these irrigation strategies improved the phenol content at “moderate” stress levels, slightly enriched the fatty acid profile (~3.5% increased oleic acid and simultaneously decreased saturated fatty acids), and increased some key volatile compounds and also several key sensory attributes. Therefore, *hydroSOStainable* olive oil may be more attractive to consumers as it is environmentally friendly, has a higher content of several bioactive compounds, and has improved sensory characteristics as compared to control (fully irrigated) oils.

## 1. Introduction

Olive trees were extended all over the Mediterranean countries by eastern civilization. Local wild trees were protected by families and tribes; thus, during many years, those olive trees that were well-adapted to environmental conditions were selected for cultivation. Consequently, olive trees are nowadays a traditional crop located in the Mediterranean basin, where originally wild olive trees existed. During the last decades, the demand for olive oil experienced a global increase; hence, it was necessary to increase its production using new intensification agronomic techniques [1]; one of these techniques was irrigation. This intensification produced an increase in tree growth and yield without affecting the quality of olive oil [1].

The three best-known olive tree varieties for super-high-density systems are “Arbequina”, “Arbosana”, and “Koroneiki”. These cultivars have fast entry into production, tend to yield good annual productions, start bearing at an early age, and have excellent oil quality characteristics [2].

In Spain, olive orchards are nowadays one of the main irrigated crops (818,505 ha), only exceeded by cereals (889,411 ha) [3]. Water scarcity is one of the main issues all over the world, and it has a clear effect on agriculture. Regulated deficit irrigation (RDI) and sustained deficit irrigation (SDI) are some of the techniques that are being developed to confront this problem. RDI decreases the use of water during some specific growing states of olives, while SDI decreases the water applied in a uniform way during all the growing season [4].

Regarding olive oil quality, the European Union and the International Olive Council have regulations to classify olive oil according to their quality [5,6]. With respect to nutritional and functional quality, it is well known that the lipid profile is one of the main contributors due to the high proportion of monounsaturated fatty acids (MUFAs) of this specific oil. Also, polyphenols have an important role, and, in fact, olive oil is the only food that has an authorized health claim [7]: “*Olive oil polyphenols contribute to the protection of blood lipids from oxidative stress*.” Volatile compounds are essential to olive oil quality, with both main and minor compounds having important roles on flavor. The odor-active compounds are responsible for the oil aroma, while the minor ones, even when they are below the olfactory threshold, can be used as quality markers as they can be essential to understand degradation or formation reactions dealing with odor-active substances [8].

*HydroSOStainable* products have been defined as fruits and vegetables cultivated under controlled deficit irrigation treatments, which give them differentiating characteristics that make them unique and environmentally friendly [9]. *HydroSOStainable* products provide special characteristics to the final commercial commodities, which are richer in some bioactive compounds and have a higher intensity of key sensory attributes, making them attractive for consumers [9,10]. Several products from olive trees under RDI are being studied as *hydroSOStainable*; for instance, “Manzanilla” table olives have been studied [11,12,13]. Arbequina olive oil under water deficit techniques have been previously studied [14,15,16,17,18], but there is not a clear trend on the effect of irrigation on its quality. At this time, there is not a systematic body of knowledge considering agronomic practices, phenological stage during RDI, climatic constraints, etc. to have a full understanding of the effects of RDI on “Arbequina” oil quality, and further studies looking for the best water saving technique are necessary.

Therefore, the aim of this work was to study the effect of (i) two new regulated deficit irrigation (RDI) treatments applied during phase II (pit hardening phase) [19] and (ii) one sustained deficit irrigation (SDI) treatment on “Arbequina” olive oil composition and properties. olive oil quality parameters (free acidity, peroxide value, and UV absorption characteristics), antioxidant, total phenol content, fatty acid profile, volatile compounds, and descriptive sensory analysis were carried out.

## 2. Results

### 2.1. Irrigation

Four irrigation treatments were applied to olive trees with different types of stress following crop water status by measuring midday stem water potential. Results of the applied water, stress integral (SI), minimum stem water potential (min ψ_stem_), yield, and mill oil yield are shown in Table 1. Not statistically significant differences in min ψ_stem_ and SI were found. Such lack of results was likely related with a wide variability of data, and min ψ_stem_ in control trees (T0) was low due to irrigation problems (for a few weeks in July). SI described better the levels of stress reached in the irrigation treatments. SI showed a tendency (*p* < 0.1). Control trees (T0) reached lower levels of stress than deficit irrigation treatments. While Confederation RDI (T2) had the highest stress (182 MPa × day) because of the reduced volume of water applied. Although Optimal RDI (T1) received a higher water volume than the Confederation SDI (T3), T1 water stress was higher because water deprivation was applied during stage II. The treatments did not affect significantly (*p* < 0.05) neither the yield, expressed in kilograms per hectare, nor the oil yield.

### 2.2. Analytical Parameters for Olive Oil Grading

Analytical parameters for olive oil grading are used to determine oil commercial quality. Following European Regulation [5], olive oil could be cataloged as extra virgin olive oil (EVOO), virgin olive oil (VOO), or lampante olive oil, which needs to be refined before consumption. EVOO has the highest quality. Results of olive oil grading are shown in Table 1. Acidity index, peroxide value, and UV absorption characteristics were under the limit established by the EU legislation; thus, it could be concluded that all oils evaluated in this study met the criteria to be categorized as EVOOs.

### 2.3. Antioxidant Activity (ABTS^+^ and DPPH^·^ Methods) and Total Polyphenols

**Figure 1:**
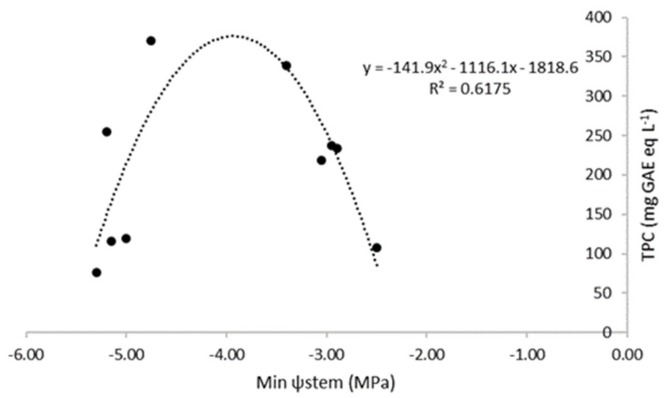
Quadratic correlation between total phenolic content (TPC (mg GAE eq L^−1^)) and minimum midday stem water potential (Min ψ_stem_ (MPa)). Data shown in this figure are the mean of 12 replications per irrigation treatment.

Results of antioxidant activity (AA), measured by two methods (ABTS^+^ and DPPH^·^), and total phenolic content (TPC) are shown in Table 1. No statistical differences between irrigation treatments were found regarding both AA methods, although a different trend was observed for TPC. Treatment 2 showed the highest value of TPC, while T1 had the smallest one. The correlation between TPC and stress level was studied, and Figure 1 shows that this correlation produced a quadratic relationship in which it could be seen that TPV increased as the minimum midday stem water potential decreased until −4 MPa; at this stress level, phenols start to decrease.

### 2.4. Fatty Acids

Fatty acids are one of the most important parameters to be analyzed in olive oil, and, in this study, 22 fatty acid methyl esters (FAMEs) were identified (Table 2), providing a very detailed characterization of the composition of the oils. Ten saturated fatty acids (SFAs) were found, with palmitic and stearic acids being the predominant ones. Regarding monounsaturated fatty acids (MUFAs), eight compounds were found, among which, oleic acid was the major one; also, the compounds C18:1 *cis*-11 and C16:1 *cis*-9 (palmitoleic acid) had important concentrations. Concerning polyunsaturated fatty acids (PUFAs), five compounds were found, standing out linoleic acid.

Irrigation treatments induced some differences among the oil composition. In general, the highest content of SFAs was found in control oils, while the smallest one was found in the Confederation SDI (T3) oils. Palmitic acid, the predominant SFA, showed this same pattern (19.93 and 19.05 g 100 g^−1^ olive oil for T0 and T3, respectively), as well as lignoceric acid. Regarding the MUFAs content, the Optimal RDI (T1) oil showed a lower value than all deficit irrigation treatments. Oleic acid was affected by the irrigation treatments, having control (47.38 g 100 g^−1^ olive oil) oils the smallest content in comparison with Optimus RDI (50.13 g 100 g^−1^ olive oil), Confederation RDI (51.29 g 100 g^-1^ olive oil), and Confederation SDI (51.00 g 100 g^−1^ olive oil). In the case of *cis*-9-heptadecenoic acid, all the oils under deficit irrigation increased their concentration in comparison to the control. Finally, and as a general finding, PUFAs were not affected by the irrigation strategies.

Results of the atherogenic index (AI) and thrombogenic index (TI) are also shown in Table 2. The oils under study had the smallest indexes compared to different oils [20], which means that olive oil is one of the most healthy oils as reflected by their low AI and TI indexes. Low AI values represent low possibilities of atheroma formation (the possibility of lipid adhesion to cells of the immune circulatory system); besides, low TI values are associated with low chances of formation of clots in the blood vessels [20].

### 2.5. Volatile Compounds

Volatile profile and composition of oils under study are shown in Table 3. Alcohols were the main chemical family found in all oils, and an increase of concentration was found in all stressed olive trees as compared to the control one. Confederation SDI (T3) was the oil with the highest alcohol content mainly due to the high contents of several compounds, including ethanol (149 mg L^-1^ olive oil), 3-methylbutan-1-ol (15.7 mg L^−1^), 2-methylbutan-1-ol (31.1 mg L^−1^), pentan-1-ol (12.0 mg L^−1^), (Z)-pent-2-en-1-ol (22.7 mg L^−1^), (Z)-hex-3-en-1-ol (303 mg L^−1^), and (E)-hex-2-en-1-ol (727 mg L^−1^ olive oil). Optimal RDI (T1) and Confederation RDI (T2) oils also showed an increase in the content of several alcohols (3-methylbutan-1-ol, 2-methylbutan-1-ol, pentan-1-ol, (Z)-hex-3-en-1-ol and (E)-hex-2-en-1-ol) as compared to the control treatment. Regarding aldehydes, the smallest concentration was found in T1 due to a decrease of pentanal, hexanal, (E)-hex-2-enal, and nonanal (0.01, 38.3, 161, and 5.86 mg L^−1^, respectively). Similarly, 2-methylbutanal and heptanal experienced a decrease in treatment T3. In general, the ketones content decreased in T1 mainly due to a reduction in the content of pentan-2-one; however, its content increased in oils T2 and T3, while pentan-3-one increased in all stressed olive trees. An intensification of the esters contents was found in T1 and T2 oils, mainly as a result of the increased contents of (Z)-hex-3-enyl acetate and hexyl acetate. Finally, a decrease in hydrocarbons was found in T1 and T3 oils, always as compared to the control, due to significant decreases in the contents of 4,8-dimethylnona-1,7-diene and (E)-4,8-dimethylnona-1,3,7-triene in the T1 oils and of (E)-β-ocimene in the T3 ones. To summarize, the highest total volatile compound contents were those of the Confederation SDI (T3) and Confederation RDI (T2) oil. Therefore, it can be concluded that *hydroSOStainable* olive oils had higher contents of volatile compounds than oil from fully irrigated trees.

### 2.6. Descriptive Sensory Analysis

After the official panel determined the commercial quality of all oils under study as EVOO, (average of 4.0 on fruity attribute), the “Food quality and safety” panel conducted descriptive sensory analysis. The lexicon and reference materials used and the sensory profiles of the studied oils are summarized in Table 4. Regarding the positive attributes of flavor, all olive oils under deficit irrigation shared a lower intensity of both green-herbs note and sourness in comparison with the control oil but increased intensities of almond and walnut notes and sweetness. In the Optimal RDI (T1), a decrease in intensity was found for most of the attributes (fruity-olive, fruity-green, floral, green-grass, and bitter). Concerning the Confederation RDI (T2) oil, fruity-olive, fruity-green, and green-herbs increased, and woody note decreased. Finally, the Confederation SDI (T3) oils also increased the intensity of the fruity-olive and woody notes but decreased that of the green-herbs note. No negative attributes (defects) were found in any of the oils under study. Concerning mouthfeel descriptors, astringency increased in T2 and T3, which could be correlated with increased polyphenol content, and, lastly, viscosity also showed an increase in the T2 and T3 oils.

In general, it can be stated that deficit irrigation during phase II of the phenological stage of “Arbequina” affected some attributes, such as fruity, green, and nuts, and the intensity of these attributes reached the highest values in the Confederation RDI (T2) oil, which experienced the highest stress. Therefore, it could be concluded that *hydroSOStainable* olive oil had a higher intensity of several key attributes than the control.

### 2.7. Pearson Correlation

In order to study the correlation between the accumulative stress in the trees and all the studied functional and sensory parameters, Pearson correlation was done with SI, and significant results are compiled in Table 5. Regarding fatty acids, a positive correlation was found between the SI and C17:1 *cis* and a negative correlation with linoleic and the total content of SFAs, meaning that the higher the stress, the better the fatty acid profile (an increase of MUFAs and decrease of SFAs). Regarding volatile compounds, a negative correlation between the SI and the aldehydes was found, but the correlation was positive for the total ester content and six compounds of this chemical family (2-methylbutanal, 2-methylbuan-1-ol, (*Z)*-hex-3-en-1-ol, (*Z)*-hex-3-enyl acetate, hexyl acetate, and (*Z)*-hex-2-enyl acetate), which are associated with increased intensity of key aroma notes, such as apple, fruity, sweet, fresh, green, and grass. Finally, green-herbs and sour showed a negative correlation with the SI, although almond, walnut, sweet, and astringency were positively correlated with the SI.

## 3. Discussion

Olive oil classification (EVOO) was not affected by RDI/SDI, in agreement with results from previous studies about water deficit irrigation on olive trees [14,18,21,22]. Description of water stress was not clear with all parameters measured and could affect some of the relationship proposed. Min ψ_stem_ was affected for variability within treatments and defined timely water stress, but presented a good agreement with some oil features. Although this measurement was not the most accurate for described irrigation treatments, it could be useful in order to describe oil features because of the reported extreme conditions. On the contrary, SI, though was also limited in comparison to irrigation treatments, it presented clear trends but did not influence the oil features. In the literature on irrigation, both indicators presented a good agreement with some yield components, such as fruit drop [23]. From our knowledge, there are very few works with the presented relationship between these water status parameter and oil features, probably because of these problems of variability.

Similar concentrations of antioxidants and TPC were reported by Sarolic et al. [24], Servili, et al. [25], and Tuberoso et al. [26] on different cultivars and also in “Arbequina” by Gomez Del Campo et al. [14] or Roodaki et al. [27], among others. In the study by Gomez Del Campo et al. [14], highest values of TPC were found when “Arbequina” olive trees were irrigated with 30% of control during the pit hardening stage, and the other irrigation treatments, even being more intense, did not show higher values. These results could be considered similar to those found in the current research, where a nonlinear relationship was found between phenolic compounds and the intensity of the water deficit. There is a previous hypothesis proposed by Horner et al. [28]: water stress in the tree can produce an increase in free phenylalanine (phenolic compounds precursor) and, therefore, phenols synthesis could be more sensitive when moderate water stress is applied.

There is contradictory information on the effect of deficit irrigation treatments on the fatty acid profile of olive oil. When stress was applied before the pit hardening stage and at the beginning of the rehydration stage, no clear effect was found neither in the study by Gucci et al. [29] nor in that of Caruso et al. [21], both with the “Frantoio” trees; these latter authors did not find a response to stress of fatty acids with a 46–48% deficit irrigation and 2–6% complementary irrigation. On the other hand, Dag et al. [30] and García et al. [22] found when studying “Koroneiki” and “Arbequina” cultivars, respectively, an increase of linoleic acid and a decrease of oleic acid, as the water stress increased during all the season. Results found by García et al. [22] (30% RDI and 60% RDI treatments before pit hardening stage) and García et al. [18] (SDI treatment with 2–3 irrigation events per week and ca. 35% of water savings and low-frequency irrigation with recovery irrigation every 3–5 weeks and ca. 35% water savings) on “Arbequina” orchard showed similar fatty acid concentrations to those of the current work; although the water stress behaved in a different way, which could be due, apart from agronomic practices, soil characteristics, climate conditions, etc., because deficit irrigation treatments were performed in a different way. Garcia et al. [22] found an increase of linolenic acid and MUFAs and a decrease of oleic acid and PUFAs in 30% RDI oils, and intermediate values for 60% RDI, while García et al. [18] found an increase of oleic acid, a decrease of linoleic acid, and MUFAs and SFAs were not affected. Therefore, it is difficult to reach a clear conclusion, considering that from the beginning of pit hardening to the end of fruit maturation, many types of enzymes contribute to synthesis of fatty acids in the olives. Irrigation has a high impact on fruit physiology, as well as the timing and the stress level [22,29]; therefore, it could be said that changes in fatty acid profiles of the studied oils in the current work could be due to the stress, but also due to the time when the stress was applied.

The synthesis of volatile compounds in olives arises during the oil accumulation phase because the main compounds (hexanal, hexyl acetate, (*Z*)-hex-3-en-1-al, (*Z*)-hex-3-en-1-ol, (*E*)-hex-2-en-1-al, (*E*)-hex-2-en-1-ol, (*Z*)-hex-3-enyl acetate, and (*Z*)-hex-2-enyl acetate) are formed through the lipoxygenase (LPO) pathway from linoleic and linolenic acids. Alcohols, esters, and ketones are also formed by fatty acid metabolism [8]. In this study, it was noticed that the alcohol concentration of all deficit irrigation oils was higher than that of the control oil. This fact may be related to an increase of LPO pathway as a result of water stress [22,25,31]. García et al. [22] found similar results to those shown in the current study; alcohols increased when water stress was applied to “Arbequina” cultivar. Other studies with different olive varieties also reported an increase of volatiles after applying water stress. It was found that 6C “green volatile” compounds, *trans*-3-hexen-1-ol, and hexyl acetate augmented when stress was applied on “Koroneiki” cultivar [31]. Similar changes on aldehydes and alcohols were reported by Servili et al. [25] on “Leccino” cultivar under water stress, as well as an increase in 2-hexen-1-ol on “Frantoio” olive oil [21]. Changes in polyphenols, volatiles, and fatty acids are directly correlated with changes in sensory descriptors of olive oil [8,32,33,34,35,36]. With respect to other cultivars under water stress, it was found that for “Leccino” and “Koroneiki” olive oils, under water stress had an increase in the pungent and bitter descriptors on oils with higher phenolic concentrations [25,31], but, with respect to “Arbequina” olive oil, it was found that deficit irrigation did not affect sensory quality [22], although Gomez Del Campo et al. [14] reported that bitterness could change when the irrigation is applied in July or August and also with the intensity of the stress. In the current study, bitterness and astringency scores only decreased in the T1 oil, where the lower concentration of polyphenols, aldehydes, and ketones was found.

## 4. Materials and Methods

### 4.1. Experimental Design and Sample Processing

Experiments were performed in 11-year-old “Arbequina” olive trees located at Carmona (37.49° N, −5.67° W, Seville, Spain). The orchard has a super-high density (4.0 m × 1.5 m), is 360 m^2^, and has 60 trees organized in 3 lines (30 m). The design was done with randomized blocks with 4 repetitions per treatment. Harvesting was done with a mechanical harvester, like at super-intensive farming. The trees from the inside row (20) of each orchard were harvested for olive oil production. Harvest was carried out when olives had 1.9 maturity index [37]. Each block was collected in one day, and the average yield was 7117 kg ha^−1^. Afterward, olive oil was elaborated in an olive mill model Frantoino Bio (Toscana Enologica Mori, Florence, Italy) at 40–50 kg h^−1^, with oil extraction 2 phases technique. Each sample milled was 100 kg of olives per plot (4 per irrigation treatment). Firstly, the olives were cleaned and washed, then they were transferred to the milling, which was held in a mill mixer (<28 °C, 20 min), with 1% (*w*:*w*) talc and 2% (*w*:*w*) water, for the extraction of water flow meter 5 L h^−1^.

Stem water potential at midday (ψ) was determined using a pressure chamber (PMS Instrument Company, Albany, OR, USA) in 4 trees per irrigation treatment, weekly during the experiment (March 24 to October 20, 2017). Water stress integral (SI) was calculated (Equation (1)) [38] to describe the accumulative effect of deficit irrigation strategies, from the beginning of pit hardening (9 June 2017) to harvest (30 October 2017) (143 days):

SI = |Σ(ψ − (−0.2)) × n|(1)

where SI is the stress integral, ψ is the average midday stem water potential for any interval, n is the number of the days in the interval.

Table 1 shows the average of minimum stem water potential (min ψ_stem_) and SI values, besides the applied water in each treatment, yield and oil yield.

Following the pressure chamber technique and the threshold values of midday stem water potential before and after the pit hardening period, 4 irrigation treatments were carried out:

- Control (T0): trees were watered to supply the 100% crop evapotranspiration (ETc).
- Optimal RDI (T1): trees were under non-limited water conditions during stage I and III while regulated deficit irrigation was applied during stage II (58% of reduction of total water irrigation amount).
- Confederation RDI (T2): the same way was followed as in T1 but with the limitation of water dotation of Guadalquivir hydrographic confederation (66% of reduction of total water irrigation amount).
- Confederation SDI (T3): sustained deficit irrigation with the water amount allowed by the Guadalquivir hydrographic confederation (66% of reduction of total water irrigation amount).

### 4.2. Analytical Parameters for Olive Oil Grading

Chemical parameters defined under EU Regulation [5] to classify the quality of olive oil were analyzed: free acidity (% of oleic acid), peroxide value (mEq O_2_ kg^−1^ oil), and UV absorption characteristics (K*_232_*, K*_270_*, and ΔK) were analyzed following the procedure described by European Union Commission [5]. UV absorption indexes were measured using cyclohexane, in a UV–visible spectrophotometer (Helios Gamma model, UVG 1002E; Helios, Cambridge, UK) and 10 mm quartz cuvettes.

### 4.3. Antioxidant Activity (ABTS^+^ and DPPH^·^ Methods) and Total Polyphenols

Measurement of antioxidants (AA) and total polyphenols (TPC) was done with an extract prepared as previously described by Tuberoso et al. [39] with some modifications. Briefly, 3 g of olive oil was mixed with 5 mL of methanol/water (80/20, *v*/*v*). The mixture was shaken for 2 min, and the hydrophilic phase was filtered with a GD/X 0.45 µm cellulose acetate septa (25 mm, Sartorius, Madrid, Spain). This procedure was repeated twice with the lipophilic phases, and all the hydrophilic extracts were evaporated in a rotary evaporator at 35 °C. Finally, the residue was dissolved in 1.5 mL of methanol.

DPPH^·^ radical (2,2-diphenyl-1-pirylhydrazyl) and ABTS^+^ (azino-bis (3-ethylbenzothiazoline-6-sulfonic acid) methods were used to evaluate the antioxidant activity (AA) of the olive oils. The DPPH^·^ was done as described by Brand-Williams et al. [40], and the ABTS^+^ as described by Re et al. [41] using a UV–visible spectrophotometer (Helios Gamma model, UVG 1002E; Helios, Cambridge, UK). Calibration curves (3.5–5.0 mmol Trolox L^−1^) with good linearity (R^2^ ≥ 0.999) were used for the quantification of the AA by both methods. Analyses were run in triplicate, and the results were expressed as mmol Trolox L^−1^ of olive oil.

Total phenolic content (TPC) was quantified using Folin-Ciocalteu reagent, as described by Gao et al. [42]. Absorbance was measured using the same extract and spectrophotometer as in AA. Gallic acid was used to prepare calibration curves. This analysis was run in triplicate, and the results were expressed as gallic acid equivalents (GAE) L^−1^ of olive oil. Gallic acid was used to facilitate comparison with previous studies.

### 4.4. Fatty Acids

Fatty acid methyl esters (FAMEs) were prepared following ISO-12966-2 [43]. The internal standard was added (C13:0; 0.04 mg mL^−1^) to calculate the fatty acids concentration. Gas chromatography (C-17A; Shimadzu Corporation, Kyoto, Japan) connected to a flame ionization detector (FID) was used to inject oils after transmethylation following ISO-12966-4 [44] with some modifications. The capillary column used was CPSil-88 (100 m × 0.25 mm ID. 0.2 µm film thickness; J&W 112-88A7; Agilent Technologies, Santa Clara, CA, USA), which is appropriate for olive oil fatty acids separation. Detector temperature was 260 °C, and oils were injected with a 1:20 split ratio. The oven temperature was 175 °C for 10 min, then raised to 220 °C (3 °C min^−1^) and kept at 220 °C for 5 min. The carrier gas was helium, and detector gases were hydrogen (30 mL min^−1^) and air (350 mL min^−1^), and helium (30 mL min^−1^) was used as a make-up gas. Standard solutions (FAME 37 MIX, Supelco; Bellefonte, PA, USA), were injected under the same conditions as oils for the identification of compounds.

Additionally, atherogenic index (AI) and thromogenic index (TI) were calculated as indicated in Equations (2) and (3) [20]:

*AI = (4 × C14:0 + C16:0) / [ΣPUFA (n - 3) + ΣPUFA (n - 6) + ΣMUFA]* (2)

where C14:0 is myristic acid, C16:0 is palmitic acid, PUFA means polyunsaturated fatty acids, and MUFA is monounsaturated fatty acids.

*TI = (C14:0 + C16:0 + C18:0) / [0.5 × ΣMUFA + 0.5 × ΣPUFA (n - 6) + 3 × ΣPUFA (n - 3) + (n - 3) / (n - 6)]* (3)

where C18:0 is stearic acid.

### 4.5. Headspace Solid-Phase Microextraction (HS-SPME)

For HS-SPME extraction, 5 mL of olive oil was added into a 15 mL glass vial with the addition of 2 µL of carvacrol (325.6 mg carvacrol in 1 L of olive oil) as an internal standard. One gram of NaCl salt was added, and the vial was hermetically sealed with polytetrafluorethilenesilicone septa and maintained in a water bath at 40 °C during equilibration (15 min) and extraction (40 min) and was partially submerged such that the liquid phase of the oils was below the water level. All the experiments were performed under constant stirring (500 rpm) with a magnetic stirrer. After sampling, the SPME fiber was inserted into the injector (250 °C) of the GC-MS for 7 min, where the extracted volatiles were thermally desorbed directly into the GC column. Polydimethylsiloxane/divinylbenzene (65 µm PDMS/DVB) fiber, obtained from Supelco Company (Bellefonte, PA, USA), was used previously conditioned according to the manufacturer instructions [24].

### 4.6. Gas Chromatography and Mass Spectrometry (GC-MS)

An Agilent Technologies (Palo Alto, CA, USA) gas chromatograph model 7890A equipped with the mass selective detector, model 5977E, and capillary column HP-5MS (5%-phenyl)-methylpolysiloxane (Agilent J & W; Santa Clara, CA, USA) GC column, 30 m, 0.25 mm i.d., coating thickness 0.25 μm was used. The flow rate of the helium carrier gas was 1.5 mL min^−1^. The injector was operated in split mode (2:1 split ratio) at 260 °C. The column was maintained at 40 °C for 3 min, heated to 100 °C at a rate of 5 °C min^−1^, heated to 260 °C at a rate of 3 °C min^−1^, and held to 260 °C for 3 min. MS conditions were as follows: source temperature 230 °C; quadrupole temperature 150 °C; transfer line temperature 270 °C; acquisition mode electron impact (EI 70 eV) by 3 scans s−1, and mass range *m*/*z* 29–350. The analyses were carried out in triplicate. The individual peaks were identified by comparison of their retention indices (relative to C9–C25 *n*-alkanes for HP-5MS) to those of authentic samples and literature as well as by comparing their mass spectra with the Wiley v9-MS library (Wiley, New York, NY, USA) and NIST14 (National Institute of Standards and Technology; Gaithersburg, MD, USA) mass spectral database [24].

### 4.7. Descriptive Sensory Analysis

Four olive oils of each irrigation treatment were analyzed by an accredited sensory panel with the purpose to determine olive oils commercial quality as described by the European regulation [5]. With that objective, oils were sent to the Laboratorio Agroalimentario de Granada (Granada, Spain) (ENAC number: 276/LE 507).

Additionally, 8 panelists from the Research Group “Food Quality and Safety” (Universidad Miguel Hernández; Alicante, Spain) analyzed the same oils to fully understand how deficit irrigation techniques affected the olive oil sensory characteristics. This panel had more than 600 h of training in sensory analysis, especially of fruit and vegetables, and it consisted of 4 males and 4 females aged from 25 to 55 years old.

The panelists did three orientation days in order to determine the scales of each attribute and the reference product. A previous lexicon developed by this panel was used [45] following International Olive Council (IOC) [46]. The scale ranged from 0 to 10, and the reference products were adapted to the Spanish market.

The descriptive sensory analysis used in this study was mandatory from the European normative to accurate oil quality [5,46], as well as other attributes to provide more detailed information about how deficit irrigation affected sensory descriptors. These descriptors were divided into 3 categories: (i) Flavor (positive attributes): fruity-olive, fruity-green, fruity-ripe, floral, green-artichoke, green-avocado, green-banana, green-herbs, green-grass, green-peppery, apple, buttery, almond, walnut, woody, piney, sweet, sour, and bitter; (ii) Flavor (negative attributes): oxidized, painty, rancid, musty, and muddy; (iii) Mouthfeel: astringent, pungent, and viscosity. Definition for all attributes and reference products with their punctuation are shown in Table 4.

### 4.8. Statistical Analyses

One-way analysis of variance (ANOVA) and Tukey’s multiple range test were performed to compare experimental data and determine significant differences among irrigation treatments (*p* < 0.05). The standard deviation (SD) of the mean is used to perform Tukey´s test; therefore, the SD values were not included in Tables to avoid repetition of the data and to make Tables easier to understand. A three-way ANOVA was used (factor 1: irrigation treatment; factor 2: session; factor 3: panelist) to study the effect of these three factors on the composition, quality, and functionality of the olive oils under study and to check panel consistency. Pearson correlation was also done to correlate all data with water stress integral. XLSTAT (Version 2016.02.27444, Addinsoft, Paris, France) was used to perform all statistical analysis.

## 5. Conclusions

It can be concluded that *hydroSOStainable* olive oils had: (i) complied with criteria to be classified as EVOO, (ii) some of them improved contents of total phenolic compounds at moderate stress levels, (iii) an enriched fatty acid profile (~3.5% increased contents of oleic acid and decreased contents of SFAs), (iv) higher contents of several volatile compounds, and (v) higher intensities of key sensory attributes, which may make them more attractive to consumers. Finally, the Confederation RDI (T2) is the recommended irrigation treatment because it (i) saved 66% of irrigation water, (ii) led to high simultaneous contents of phenolic compounds and slightly increased the monounsaturated fatty acids, and (iii) had a balanced sensory profile.

## Figures and Tables

**Table 1:** Watering technique conditions, oil technological parameters, antioxidant activity (ABTS^+^ and DPPH^·^ methods), and total phenol content (TPC) of “Arbequina” olive oil.

	**ANOVA^†^**	**T0**	**T1**	**T2**	**T3**
**Watering Technique Conditions**					
Applied water (mm)		468	197	160	162
Stress integral (MPa × day)	NS†	53.4	152	182	132
Min ψ_stem_ (MPa)	NS	−3.80	−4.00	−4.68	−4.04
Yield (kg ha^−1^)	NS	7287	6902	6316	6764
Oil Yield (% dry weight)	NS	28.0	30.4	30.1	33.0
**Olive Oil Quality Parameters**					
Acidity index (%)	NS	0.31	0.37	0.24	0.31
Peroxide value (meq O_2_ kg^−1^)	NS	9.29	8.07	9.36	10.1
K*_232_*	NS	2.15	1.91	2.14	2.02
K*_270_*	NS	0.10	0.10	0.11	0.10
ΔK	NS	−0.03	−0.02	−0.02	−0.02
**Antioxidant Activity and Total Phenol Content**					
ABTS^+^ (mmol Trolox eq L^−1^)	NS	0.113	0.098	0.114	0.151
DPPH (mmol Trolox eq L^−1^)	NS	0.233	0.223	0.265	0.282
TPC (mg GAE L^−1^)	*	259.8 a^‡^	126.8 b	267.3 a	181.5 ab

† NS = not significant at *p* < 0.05; *, significant at *p* < 0.05. ‡ Values of olive oil quality parameters, antioxidant activity, and total phenolic content (TPC) (mean of 12 replications per irrigation treatments) followed by the same letter, within the same row, were not significantly different (*p* < 0.05), according to Tukey’s least significant difference test. Note: Acidity index: Threshold value for extra virgin olive oil (EVOO) is ≤0.8%; peroxide value: threshold value for EVOO is ≤20 meq O_2_ kg^−1^; K*_232_*: threshold value for EVOO is ≤2.5; K*_270_*: threshold value for EVOO is ≤0.22; ΔK: threshold value for EVOO is ≤0.01; (EEC Regulation 2568/91). T0: control (100% ETc); T1: Optimal RDI (RDI during stage II); T2: Confederation RDI (RDI during stage II using water limitation of Guadalquivir hydrographic confederation); T3: Confederation SDI (SDI using water limitation of Guadalquivir hydrographic confederation). ABTS^+^: azino-bis (3-ethylbenzothiazoline-6-sulfonic acid; DPPH^·^ 2,2-diphenyl-1-pirylhydrazyl.

**Table 2:** Fatty acid profiles of “Arbequina” olive oil as affected by the irrigation treatment.

	Compound	Concentration (g 100 g^−1^ Olive Oil)				
		ANOVA^†^	T0	T1	T2	T3
1	Tetradecanoic acid (Myristic acid)	NS	0.025	0.024	0.022	0.025
2	Pentadecanoic acid	NS	0.016	0.018	0.019	0.017
3	Hexadecanoic acid (Palmitic acid)	*	19.93 a^‡^	19.06 b	18.96 b	19.05 b
4	*cis*-6-Hexadecenoic acid (Sapienic acid)	NS	0.207	0.206	0.196	0.202
5	*cis*-9-Hexadecenoic acid (Palmitoleic acid)	NS	3.624	3.254	3.292	3.071
6	*cis*-11-Hexadecenoic acid	NS	0.030	0.025	0.023	0.021
7	Heptadecanoic acid (Margaric acid)	NS	0.135	0.150	0.161	0.154
8	*cis-9-*Heptadecenoic acid	*	0.279 b	0.310 a	0.324 a	0.312 ab
9	Octadecanoic acid (Stearic acid)	*	1.880 b	1.970 a	2.039 a	2.052 a
10	*trans*-9-Octadecenoic acid (Eleaidic acid)	NS	0.013	0.020	0.015	0.016
11	*cis*-9-Octadecenoic acid (Oleic acid)	**	47.38 b	50.13 a	51.29 a	51.00 a
12	*cis*-11-Octadecenoic acid	NS	7.026	6.514	6.537	6.419
13	9,12-Octadecadienoic acid (Linoleaidic acid)	NS	0.032	0.031	0.027	0.029
14	9,12-Octadecadienoic acid (Linoleic acid)	NS	17.55	15.90	14.76	15.38
15	Eicosanoic acid (Arachidic acid)	NS	0.512	0.499	0.497	0.506
16	6,9,12-Octadecatrienoic acid (γ-linolenic acid)	NS	0.007	0.010	0.010	0.008
17	*cis*-11-Eicosenoic acid (Gondoic acid)	NS	0.333	0.341	0.339	0.341
18	9,12,15-Octadecatrienoic acid (α-linolenic acid	NS	0.902	0.866	0.799	0.794
19	Heneicosanoic acid	NS	0.014	0.015	0.014	0.015
20	Docosanoic acid (Behenic acid)	NS	0.159	0.155	0.154	0.161
21	Tricosanoic acid	NS	0.043	0.040	0.040	0.035
22	Tetracosanoic acid (Lignoceric acid)	*	0.101 a	0.091 b	0.090 b	0.091 b
Σ SFAs		NS	22.29	22.00	21.98	22.09
Σ MUFAs		**	59.78 b	61.66 a	62.81 a	62.17 a
Σ PUFAs		NS	17.61	15.96	14.82	15.43
Atherogenic index, AI		NS	0.326	0.311	0.303	0.308
Thrombogenic index, TI		NS	0.520	0.513	0.515	0.517

^†^ NS = not significant at *p* < 0.05; *, **, significant at <0.05 and 0.01, respectively. ^‡^ Values (mean of 12 replications per irrigation treatment) followed by the same letter, within the same row, were not significantly different (*p* < 0.05), according to Tukey’s least significant difference test. Note: SFAs: saturated fatty acids; MUFAs: monounsaturated fatty acids; PUFAs: polyunsaturated fatty acids.

**Table 3:** Volatile profile (polydimethylsiloxane/divinylbenzene (PDMS/DVB) fiber) of “Arbequina” olive oils as affected by irrigation treatment.

	RI^¥^	Compound	Sensory Descriptor	Concentration (mg L^−1^ Olive Oil)				
				ANOVA^†^	T0	T1	T2	T3
V1	<500	Ethanol	Alcohol, apple, sweet	*	56.3 b^‡^	51.0 b	54.7 b	149 a
V2	568	Ethyl acetate	Aromatic, bitter, fruity	*	11.0 b	13.7 b	0.00 c	41.4 a
V3	609	Pentanal	Nutty, fruity, vanilla	*	12.8 a	0.01 c	9.68 b	11.2 ab
V4	659	2-Methylbutanal	Apple, fruity, ripe	**	7.00 b	8.93 b	17.1 a	0.01 c
V5	677	Pent-1-en-3-ol	Butter, fruity, green	*	19.7 c	17.0 c	32.5 a	26.0 b
V6	684	Pentan-2-one	Fruity, apple, pineapple	**	30.8 b	26.9 c	41.4 a	36.3 ab
V7	697	Pentan-3-one	Bitter, green, mustard	*	30.1 c	34.1 bc	39.7 b	49.2 a
V8	726	3-Methylbutan-1-ol	Sweet, woody, yeast	***	10.2 c	12.1 b	11.3 b	15.7 a
V9	730	2-Methylbutan-1-ol	Winey, spicy	*	14.0 c	20.6 b	21.3 b	31.1 a
V10	757	Pentan-1-ol	Balsamic, fruity, pungent	*	5.52 c	8.07 b	9.30 b	12.0 a
V11	762	(*Z*)-Pent-2-en-1-ol	Almond, banana, fruity	**	10.5 b	13.2 b	12.3 b	22.7 a
V12	799	Hexanal	Apple, banana, grass, green	***	63.1 b	38.3 c	65.9 b	87.3 a
V13	848	(*E*)-Hex-2-enal	Almond, apple, astringent	***	373 a	161 c	237 b	187 bc
V14	851	(*Z*)-Hex-3-en-1-ol	Apple, banana, fresh, grass	***	198 b	285 ab	279 ab	303 a
V15	861	(*E*)-Hex-2-en-1-ol	Apple, flowers, fruity, grass	*	237 b	362 ab	360 ab	727 a
V16	863	Hexan-1-ol	Banana, fruity, soft, tomato	NS	388	397	345	368
V17	890	Heptan-2-one	Banana, cinnamon, fruity	NS	4.51	1.22	3.07	0.00
V18	898	2-propenylcyclopentane		NS	9.01	4.47	14.2	8.27
V19	904	Heptanal		*	10.0 ab	12.2 ab	16.6 a	8.48 b
V20	935	3-Ethylocta-1,5-diene (isomer 1)		*	25.4 ab	19.8 b	28.5 a	28.6 a
V21	942	3-Ethylocta-1,5-diene (isomer 2)		*	26.7 ab	18.6 b	28.5 a	28.3 a
V22	998	4,8-dimethylnona-1,7-diene		**	45.8 a	27.7 b	48.7 a	42.3 a
V23	1007	(*Z*)-Hex-3-enyl acetate	Green, banana	***	229 b	377 a	357 a	236 b
V24	1016	Hexyl acetate	Green, fruity, sweet	*	70.7 c	112 a	116 a	103 b
V25	1019	(*Z*)-Hex-2-enyl acetate	Apple, banana, grape	***	8.41 a	8.35 a	8.85 a	0.87 b
V26	1053	(*E*)-β-Ocimene	Sweet, herbal	*	22.7 a	10.3 ab	8.96 ab	6.61 b
V27	1098	Methyl benzoate	Fruity	**	5.65 a	0.21 b	0.01 b	0.87 b
V28	1107	Nonanal	Apple, coconut, grape	*	12.7 a	5.86 b	10.6 ab	8.51 ab
V29	1120	(*E*)-4,8-Dimethylnona-1,3,7-triene	-	*	14.4 a	9.25 b	13.2 ab	12.9 ab
V30	1208	Methylcyclodecane	-	NS	17.3	7.85	12.7	11.8
		Σ Alcohols		***	938 b	1165 ab	1124 ab	1654 a
		Σ Aldehydes		***	478 a	226 b	356 ab	302 ab
		Σ Ketones		**	143 ab	112 b	167 a	162 a
		Σ Esters		***	324 b	511 a	482 a	382 b
		Σ Hydrocarbons		**	82.9 a	47.3 c	70.8 ab	61.8 b
		Σ Volatile compounds		*	1966 b	2061 b	2200 ab	2562 a

^†^ NS = not significant at *p* < 0.05; *, **, ***, significant at *p* < 0.05, 0.01, and 0.001, respectively. ^‡^ Values (mean of 12 replications per irrigation treatment) followed by the same letter, within the same row, were not significantly different (*p* < 0.05), according to Tukey’s least significant difference test. ^¥^ Retention index.

**Table 4:** Descriptive sensory profiles of “Arbequina” olive oil as affected by the irrigation treatment.

	Descriptor	References	ANOVA^†^	T0	T1	T2	T3
Flavor (positive attributes)							
**D1**	Fruity-olive	Canned Ripe Olives, Pitted Black = 2.3 Hacendado, Manzanilla Green olives = 5.3	***	3.9 ab^‡^	3.3 b	4.2 a	4.3 a
**D2**	Fruity-green (under-ripe olive)	Canned Ripe Olives, Pitted Black = 1.0 Hacendado, Manzanilla Green olives = 2.7	*	2.6 ab	2.2 b	3.0 a	2.6 ab
**D3**	Fruity-ripe (ripe olive)	Canned, Ripe Olives, Pitted Black = 1.0 Hacendado, Manzanilla Green olives = 3.7	NS	1.50	1.75	1.63	1.75
**D4**	Floral	Pompadour, Chamomile Herbal Tea = 5.0 Carrefour, White Grape Juice (diluted 1:1) = 4.7	*	1.3 a	0..8 b	1.2 a	1.3 a
**D5**	Green-artichoke	Hacendado, Artichoke Hearts = 3.0	NS	0.8	0.5	0.6	0.7
**D6**	Green-avocado	Under-ripe Fresh Avocado = 5.3	NS	0.5	0.5	0.5	0.6
**D7**	Green-banana	Under-ripe Green Banana = 4.0	NS	0.40	0.38	0.34	0.31
**D8**	Green-herbs	Verdifresh Arugula (organic, washed) = 5.7	*	2.2 a	1.3 b	1.6 b	1.6 b
**D9**	Green-grass	*Cis*-3-Hexen-1-ol 1000 ppm= 10.0	*	1.3 ab	0.8 b	1.5 a	0.9 b
**D10**	Green-peppery	Hacendado, Green-Peppercorns (dried) = 2.0	NS	0.6	0.5	0.6	0.5
**D11**	Apple	Fuji Apple = 5.0	NS	0.1	0.	0.21	0.4
**D12**	Buttery	Under-ripe Fresh Avocado = 4.0	NS	0.9	0.7	0.7	0.9
**D13**	Almond	Hacendado, almonds = 5.0	*	0.3 b	0.4 a	0.4 a	0.5 a
**D14**	Walnut	Hacendado, walnuts = 6.0	*	0.2 b	0.5 a	0.4 a	0.4 a
**D15**	Woody	Hacendado, walnuts = 3.0	*	0.4 ab	0.5 a	0.4 b	0.6 a
**D16**	Piney	Hacendado, pine nuts = 3.5	NS	0.4	0.5	0.5	0.5
**D17**	Sweet	1% sucrose solution = 3.0	*	0.8 b	1.4 a	1.3 a	1.4 a
**D18**	Sour	0.05% citric solution = 2.5	**	0.8 a	0.4 b	0.6 b	0.6 b
**D19**	Bitter	0,01% caffeine solution = 1.0	**	0.8 a	0.5 b	0.7 a	0.9 a
Flavor (negative attributes)							
**D20**	Oxidized	La Masía, 100% sunflower oil ^a^ = 4.0	NS	0.00	0.00	0.00	0.00
**D21**	Painty	Hacendado, Green-Peppercorns (dried) = 3.3	NS	0.00	0.00	0.00	0.00
**D22**	Rancid	International olive council standard = 9.2	NS	0.00	0.00	0.00	0.00
**D23**	Musty	International olive council standard = 4.65	NS	0.00	0.00	0.00	0.00
**D24**	Muddy	International olive council standard = 7.9	NS	0.00	0.00	0.00	0.00
Mouthfeel							
**D25**	Astringent	0,10% alum solution = 4.0	***	0.9 b	0.7 b	1.9 a	1.2 ab
**D26**	Pungent	Verdifresh Arugula (organic, washed) = 5.0	NS	2.7	2.5	2.6	2.9
**D27**	Viscosity	Hacendado, condensed milk = 10.0	***	3.9 b	3.3 b	4.2 a	4.2 a

^†^ NS = not significant at *p* < 0.05; *, **, ***, significant at *p* < 0.05, 0.01, and 0.001, respectively. ^‡^ Values (mean of 12 replications per irrigation treatment) followed by the same letter, within the same row, were not significantly different (*p* < 0.05), according to Tukey’s least significant difference test.

**Table 5:** Pearson correlation between Stress Integral (SI) and fatty acids, volatile compounds, and descriptive sensory analysis attributes.

	SI
Fatty Acids	
C17:1 *cis*	0.546^†^*
Linoleic (C18:2 *cis*)	−0.568*
SFAs	−0.562*
Volatile Compounds	
2-Methylbutanal	0.657**
2-Methylbutan-1-ol	0.559*
(Z)-Hex-3-en-1-ol	0.670**
(Z)-Hex-3-enyl acetate	0.778**
Hexyl acetate	0.729**
(Z)-Hex-2-enyl acetate	0.602*
Σ Aldehydes	−0.706**
Σ Esters	0.871***
Descriptive Sensory Analysis	
Green-herbs	−0.841***
Almond	0.834***
Walnut	0.811***
Sweet	0.881***
Sour	−0.849***
Astringent	0.603*

^†^*, ** and ***, significant at *p* < 0.05, 0.01, and 0.001, respectively.
